# *Centaurea* Subsect. *Phalolepis* (Compositae, Cardueae): A Case Study of Mountain-Driven Allopatric Speciation in the Mediterranean Peninsulas

**DOI:** 10.3390/plants12010011

**Published:** 2022-12-20

**Authors:** Núria Garcia-Jacas, Jordi López-Pujol, Neus Nualart, Sonia Herrando-Moraira, Konstantin Romaschenko, Ming-Xun Ren, Alfonso Susanna

**Affiliations:** 1 Botanic Institute of Barcelona (IBB, CSIC-Ajuntament de Barcelona), Pg. Migdia, s/n, 08038 Barcelona, Spain; 2 Escuela de Ciencias Ambientales, Universidad Espíritu Santo (UEES), Samborondón 091650, Ecuador; 3 Department of Botany, National Museum of Natural History, Smithsonian Institution, Washington, DC 20013-7012, USA; 4 M.G. Kholodny Institute of Botany, National Academy of Sciences of Ukraine, 01601 Kyiv, Ukraine; 5 Key Laboratory of Genetics and Germplasm Innovation of Tropical Special Forest Trees and Ornamental Plants (Hainan University), Ministry of Education, Haikou 570228, China; 6 Center for Terrestrial Biodiversity of the South China Sea, Hainan University, Haikou 570228, China

**Keywords:** genetic diversity, allopatric speciation, ruggedness, climate stability

## Abstract

*Centaurea* subsection *Phalolepis* has been thoroughly analyzed in previous studies using microsatellites in four centers of speciation: Anatolia, Greece, the Italian Peninsula and the Iberian Peninsula. Evidence suggests a correlation between taxon diversity and mountains. This group constituted a good case study for examining the mountain–geobiodiversity hypothesis (MGH), which explains the possible reasons for the many radiations occurring in mountains across the world. We combined all the datasets and carried out analyses of their genetic structure to confirm the species of subsect. *Phalolepis* are grouped according to a geographic pattern. We then checked whether climatic fluctuations favored the “species pump” hypothesis in the mountains by using the Climatic Stability Index (CSI). Finally, the relief of the terrain was tested against the rate of allopatric speciation by region by means of Terrain Ruggedness Index and environmental gradients through our new Climate Niche Breadth Index. Our results supported the MGH hypothesis and confirmed that the main triggers, namely altitudinal zonation, climatic oscillations and rugged terrain, must be present for the development of a radiation.

## 1. Introduction

Mountains are often linked to high levels of biodiversity [1,2]. In recent times, a study focused on the Tibeto-Himalayan region (THR) led to the definition of the “mountain–geobiodiversity hypothesis” or MGH to explain its species richness [3]. These authors proposed that three conditions are required to maximize the impact of mountain formations and surface uplift on regional biodiversity patterns, which are key to the origin of montane biodiversity. These are (i) complete altitudinal zoning with lowland, montane and alpine zones; (ii) climatic fluctuations that enable mountains to act as “species pumps”; and (iii) high-relief terrain with large environmental gradients that favor both species persistence in suitable refugia and allopatric speciation driven by geographic barriers. This hypothesis is very attractive and fits partly our own results in the Tibet–Himalaya–Hengduan region for the genus *Saussurea* (Herrando et al., pers.comm.). However, extrapolation of this hypothesis to other mountain systems remains unclear, despite the claims about this extrapolation made by [4].

In the Mediterranean environment, we do not find extremely rugged mountainous regions, but there might be some with the conditions proposed by [3] needed for high levels of biodiversity to occur. For example, Tahtali Dag is a mountain 2300 m high situated in south Turkey only 10 km from the sea. These rugged and diverse regions generally coincide with biodiversity hotspots that, in turn, coincide with refuges of plant diversity during the Ice Ages [5]. For example, the climatic fluctuations of the Quaternary Period led to cycles of connection and isolation of species, and the presence of relief with environmental gradients provided the necessary isolation for allopatric speciation, increasing the number of species and endemics in these areas [6,7]. In the opposite case, when there is no relief, speciation is often hybridogenic with continuous contact among species through hybridization and gene flow.

Despite the interest in the subject, there have been no studies covering the whole of the Mediterranean Basin; research has concentrated on local altitudinal studies and microrelief [8,9]. We focused our study on a wide environment, namely the mountains of the south of the Mediterranean region, by using the *Phalolepis* subsection of the genus *Centaurea* as a model. This group of ~40 species has already been used as a model in a series of studies exploring the correlation between genetic data and taxonomy [10,11,12,13] that emphasized the importance of the mountains in the speciation of the group. *Phalolepis* has diversified in several nuclei that coincide with some of the main hotspots and refuges [5] of the Mediterranean Region: Anatolia, South Balkans (Greece), the Italian Peninsula and the Iberian Peninsula. The southern peninsulas of Europe became important refugia for species throughout the Ice Ages [14], as reflected in their rich flora. Here, we briefly describe these four Mediterranean refugia.

The Anatolian Peninsula is a crossroad of two floristic domains: the Irano-Turanian and Mediterranean. The Mediterranean floristic domain is confined to the western and southern coastal strips and adjacent mountain ranges, mainly the Western and Central Taurus, with some minor enclaves on the coast of the Black Sea. The Central Anatolian plateau and the eastern part of the peninsula is dominated by the Irano-Turanian vegetation [15]. The mountain ranges in Anatolia were a refugium for Mediterranean species during periods of glaciation, the main specific refugia being the Amanus, Western Anatolia and the Taurus. The species richness is extreme: the Mediterranean part of the Anatolian Peninsula treasures ~5000 species, 30% of them endemic [6].

Mainland Greece is extremely mountainous; specifically, central and western Greece is divided by several irregular, abrupt mountain ranges such as the Pindus and the Olympus massif [16]. Climatic fluctuations during Pleistocene glaciations resulted in widespread changes in the vegetation [17], while the combination of climatic fluctuations and rugged terrain favored the expansion of some species. Subsequent isolation resulted in the allopatric speciation of many local endemics, which are usually montane species growing above 600 m [18]. This fact highlights the role of mountains as centers of speciation [9,19].

The Italian Peninsula is also mountainous, with the Apennines chain running throughout the peninsula from the Alps to the extreme south (Calabria). However, the northern and central Apennines do not belong to the Mediterranean domain. As well as the widespread *Centaurea deusta*, all the *Centaurea* species of subsect. *Phalolepis* described in Italy are concentrated in the southern Apennines and especially in the Calabrian mountains located on the southern tip of the Italian Peninsula [12,20]. This region was an important refugium during the Ice Ages [7].

The Iberian Peninsula is a region with less mountainous topography. Despite the presence of mountain ranges arranged generally in an east–west direction (Pyrenees, Cantabrian Mountains, and the Iberian, Central and Penibaetic ranges), most of the territory is occupied by a 600 m high plateau, the Meseta. It constitutes a wide area that is open for migrations and expansions that impede isolation in the mountains, as verified in other studies on *Centaurea* [13,21].

The tentative conclusion of the four studies carried out on the *Phalolepis* subsect. of *Centaurea* [10,11,12,13] is that the correlations among the number of species, genetic data and classification reached their maximum in the two regions with the most rugged topography, namely Anatolia and Greece. In these two regions, the number of endemic *Centaurea* species is the highest, and the studies confirmed that they are genetically well-defined species [10,11]. The number of species decreases in an east–west gradient as the roughness of the mountains diminishes: in the Italian Peninsula, genetic studies do not support the existence of the microspecies described in the south of the peninsula [12]. As for the Iberian Peninsula, our study ruled out the presence of allopatric speciation and suggested that all speciation events in the group occurred via hybridization [13].

With this background, the most consistent hypothesis is that the southern Mediterranean mountains have promoted allopatric speciation in *Phalolepis*, favoring population isolation and allopatry, likely following the MGH model [3]. To test this hypothesis, we pooled the genetic data obtained for the same set of microsatellites from the four *Phalolepis* speciation centers and conducted a combined analysis to confirm that the studied taxa are genetically arranged following the geographic speciation centers. After verifying this premise, we next examined whether the current diversity of species of *Phalolepis* in the four Mediterranean peninsulas agreed with a MGH model. The first condition (the presence of lowland, montane and alpine zones) is met in the four speciation centers, and thereafter, we focused on the second and third conditions of this model. To explore the second condition, i.e., the presence of climatic fluctuations that enabled mountains to act as “species pump”, we used the recently published Climatic Stability Index (CSI) [22] to examine whether there is a correlation between climatic instability and the impetus of allopatric speciation. Finally, we examined the components of the third condition, namely high relief terrain (ruggedness) and environmental gradients (climate niche breadth), which were also tested against the rate of allopatric speciation by region.

## 2. Material and Methods

### 2.1. Plant Material

**Figure 1:**
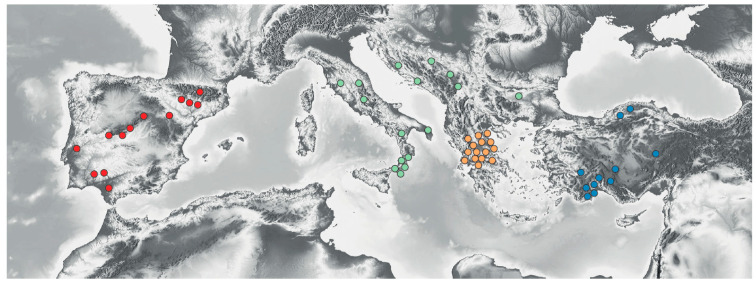
Map of the Mediterranean region, showing the geographic distribution of the sampled populations. In red, populations from the Iberian Peninsula; in green, populations from the Italian Peninsula and Northern Balkans; in cream, populations from Greece; in blue, populations from Turkey. The base map was downloaded from https://maps-for-free.com/ (accessed 23 June 2022).

The total number of individuals studied was 1487 from 56 populations belonging to 21 species, all of them diploid (Figure 1). The number of species by region was as follows: Iberian Peninsula, two species (*C. alba* L. and *C. costae* Willk.); Italian Peninsula + Northern Balkans, six species (*C. aspromontana* Brullo, Scelsi & Spamp., *C. ionica* Brullo, *C. nobilis* (Groves) Brullo, *C. pentadactyli* Brullo, Scelsi & Spamp., *C. scillae* Brullo and *C. deusta* Ten.); Greece, seven species (*C. brunnea* (Halácsy) Halácsy, *C. chrysocephala* Phitos & T. Georgiadis, *C. heldreichii* Halácsy, *C. litochorea* T. Georgiadis & Phitos, *C. messenicolasiana* T. Georgiadis, Dimitrellos & Routsi, *C. princeps* Boiss. & Heldr. and *C. deusta*); Turkey, seven species (*C. amaena* Boiss. & Balansa, *C. antalyensis* H. Duman & A. Duran, *C. cadmea* Boiss., *C. luschaniana* Heimerl ex Stapf, *C. lycaonica* Boiss. & Heldr., *C. lycia* Boiss., and *C. wagenitzii* Hub.-Mor.). *Centaurea deusta* and *C. alba* are species with a wide distribution compared with the rest, which are narrow endemics. The voucher information is shown in Supplemental Table S1. The Italian Peninsula and the Northern Balkans were merged in a single unit for this analysis because of the evidence of recent connections between both regions [23].

### 2.2. Genetic Analyses

#### 2.2.1. Microsatellite Loci Data

For our analyses, we used the results obtained for the four geographic regions for the same seven amplified microsatellite loci (*CD37*, *42CM27*, *12B1*, *13D10*, *17E3*, *21D9* and *28A7*) in our previous works [10,11,12,13]. All these SSR were originally developed for *C. corymbosa* Pourr., *C. diffusa* Lam. and *C. stoebe* L. [24,25].

#### 2.2.2. Genetic Structure and Allopatric Speciation Parameters

For the analysis of the genetic structure, the clustering software Structure v. 2.3.4 [26], which is based on a Bayesian approach, was used. In total, 1487 individuals (from 56 populations from the four regions) were analyzed. After a preliminary run with the number of clusters (*K*) set from 1 to 56 and 10 iterations per *K*, we restricted *K* to 1 to 37 and increased the number iterations per *K* to 20, assuming an admixture model with correlated allele frequencies and no “locprior”. The burn-in length and the number of Markov chain Monte Carlo (MCMC) replications were set to 10^5^ and 10^6^, respectively. The most likely value of *K* was determined in two ways: by choosing the smallest *K* according to the log-probability of the data (ln Pr(*X*|*K*)) where the values reached a plateau [27], and by the Δ*K* statistical approach of [28] implemented in the software Structure Harvester v. 0.6.94 [29]. Finally, the programs Clumpp v. 1.1.2 [30] and Distruct v. 1.1 [31] were used in order to obtain a clear and interpretable image of this analysis. Additionally, the software GenAlEx v. 6.5 [32] was used to perform a principal coordinate analysis (PCoA) at the population level, based on the codominant genotype distances.

To infer the degree of allopatric speciation in *Centaurea* subsect. *Phalolepis*, we compared the levels of genetic diversity, genetic diversification, recent gene flow and past gene flow for the four speciation areas [10,11,12,13]. Specifically, we calculated the expected heterozygosity (*H*_e_) as the best estimator of genetic diversity [33], using GenAlEx. For genetic differentiation between populations, we chose *F*_ST,_ which was calculated using FreeNA [34] and/or Arlequin v. 3.11 [35]. For recent gene flow, we used recent (last one–three generations) migration rates (*m*) estimated using the program BayesAss v. 1.3 and v. 3.0 [36]. For older rates of gene flow, we calculated historical mutation-scaled migration rates (*M*), originally obtained with the software MIGRATE-N [37]. The effective number of migrants per generation (*Nm*) was estimated using the formula 4*Nm* = Θ*M* [38]. For more explanations and descriptions of the genetic parameters and the methods used to estimate them, please refer to the four cited studies.

### 2.3. Testing the MGH Model

To check whether the second and third conditions (climatic fluctuations and high-relief terrain with large environmental gradients, respectively) of the MGH model were responsible for the higher degree of allopatric speciation of the eastern compared with western speciation centers, we calculated the values for adequate surrogates of three conditions and we searched for statistical differences among the four geographic regions. For the second condition, we chose the Climatic Stability Index (CSI) [22]. The “CSI-past” uses 14 of the 19 bioclimatic variables of the WorldClim database (https://www.worldclim.org, accessed 20 November 2020) for the 12 periods of PaleoClim [39] that represent warm/cold cycles from the Pliocene (ca. 3.3 Ma) to the present; it is available at a 2.5 arc-min (~5 km) resolution covering the whole planet, with values between 0 (completely stable areas) and 1 (the most unstable ones). We believe that the CSI is particularly suited for our species system because speciation events within *Centaurea* subsection *Phalolepis* took place from the mid-Pliocene to the present [40,41] within the timespan covered by the CSI. The CSI was clipped to an area delimited by 50° N, 12° W, 33° N and 42° E in R using the package Raster v.3.5-15 [42].

For the third condition, we chose one variable for each of the two subconditions. For representing high-relief terrain, we chose the terrain ruggedness index (TRI), which is the mean of the absolute differences in elevation between a focal cell and its eight surrounding cells; flat areas have a value of zero. The TRI was obtained from [43], and it is available at several resolutions (we used a 2.5 arc-min resolution). For the second subcondition (environmental gradients), we generated an ad hoc variable, the Climate Niche Breadth (CNB), which was estimated by using all 19 bioclimatic variables (at a 30 arc-sec resolution) of the WorldClim database (accessed on 28 February 2022). The CNB was calculated by using the R package base v.4.1.1 [44] as follows. First, we calculated the standard deviation (SD) for each bioclimatic variable selected (see below) from the 25 pixels at 30 arc-sec resolution that were included within a 2.5 arc-min pixel. We then normalized all the values for each 2.5 arc-min pixel between 0 and 1. Finally, we summed all the SD values for each selected variable (see below). Higher CNB values represented broad (climate) niches, whereas lower values indicated narrow niches. Both the TRI and CNB were clipped to the same study area as the CSI.

As there could be problems associated with the collinearity of bioclimatic variables, we performed a variable selection process to reduce the autocorrelation effect for estimating both the CSI and CNB. With the clipped variables confined to the study area, a Pearson correlation analysis was performed to identify the possibly highly correlated variable sets using the R package Stats v.4.1.1 [44]. Next, a principal component analysis (PCA) was conducted by using the R package FactoMineR v.2.4 [45] to reduce the dimensions of the explanatory variables. The contribution of the variables to each of the PCA axes was obtained with the R package factoextra v.1.0.7. [46]. We retained the first three principal components (PCs) that accounted for >80% of the variance in the original data [47]. The three variables that contributed the most to explaining variance in the first three PCs were conserved; of these, we discarded those with the least percentage of contribution to that axis from highly correlated sets (Pearson’s *r* >|0.9|). For the CSI, six final variables were used for further analysis: bio8 (the mean temperature of the wettest quarter), bio11 (the mean temperature of the coldest quarter), bio16 (the precipitation of the wettest quarter), bio17 (the precipitation of the driest quarter), bio18 (the precipitation of the warmest quarter) and bio19 (the precipitation of the coldest quarter). For the CNB, the variables selected, in addition to bio8, bio17 and bio19, were bio5 (the maximum temperature of the warmest month), bio6 (the minimum temperature of the coldest month) and bio15 (seasonality of precipitation).

The values for the three variables (CSI, TRI and CNB) for each of the speciation centers were calculated by three different strategies: (1) using only the species occurrence points, i.e., the values for the three variables were extracted for each occurrence with the “extract” function of the raster package in R and (2) delimiting 10 km buffer areas around each occurrence. Comparisons among the speciation centers were statistically tested. First, the Shapiro–Wilk test was performed to check the normality of the data; as they were not normally distributed (the *p* value was less than an alpha level of 0.05), non-parametric Wilcoxon–Mann–Whitney analysis was conducted. The analyses were performed using the shapiro.test and wilcox.test functions of the package Stats v. 4.1.1 in R.

## 3. Results

### 3.1. Genetic Structure and Allopatric Speciation Parameters

**Figure 2:**
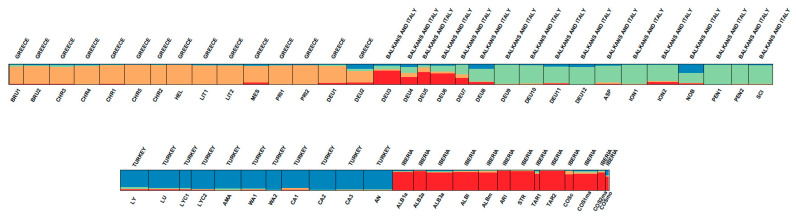
Membership of 1487 individuals from 56 populations of 21 species within four groups (*K* = 4) according to the Bayesian analysis of the population structure carried out with Structure.

**Figure 3:**
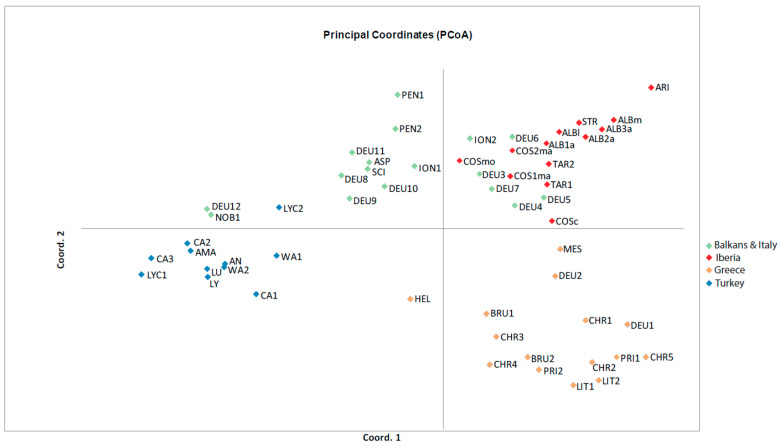
Principal coordinate analysis performed from pairwise genetic distances between populations. For the abbreviations of the populations, see Supplementary Table S1.

According to the approach of [28] for calculating the best number of clusters (*K*), the most likely value of *K* was 4 (Supplementary Figure S1), in agreement with the four biogeographic areas of subsection *Phalolepis*: Greece (cream color in Figure 2), Turkey (blue), the Iberian Peninsula (red) and Italy and the Northern Balkans (green), although this last region showed a high degree of admixture, specifically in the Northern Balkans (Figure 2). In the principal coordinate analysis (PCoA) at population level (Figure 3), the resolution was good as far as the Greek and Turkish populations were concerned. However, the Balkan and Italian populations ION2, DEU3, DEU4, DEU5, DEU6 and DEU7 showed a clear overlapping zone with the Iberian populations in Figure 3; in these six populations, the presence of the Iberian genetic cluster (indicated by the red color in Figure 2) was evident in the Structure analysis.

Regarding the genetic diversity, the Iberian Peninsula and Northern Balkans–Italy were the areas with higher genetic variation (measured as the expected heterozygosity *H*_e_) and both past and recent gene flow (Table 1). In particular, the Iberian Peninsula had clearly much higher values (about one order of magnitude higher) for both recent and past gene flow than the other areas. For genetic differentiation (*F*_ST_), the values from our previous analyses were the highest in Greece and lowest in the Iberian Peninsula (*F*_ST_ = 0.243 and 0.176, respectively; see Table 1).

### 3.2. Testing the Mountain–Geobiodiversity Hypothesis (MGH) Model

**Figure 4:**
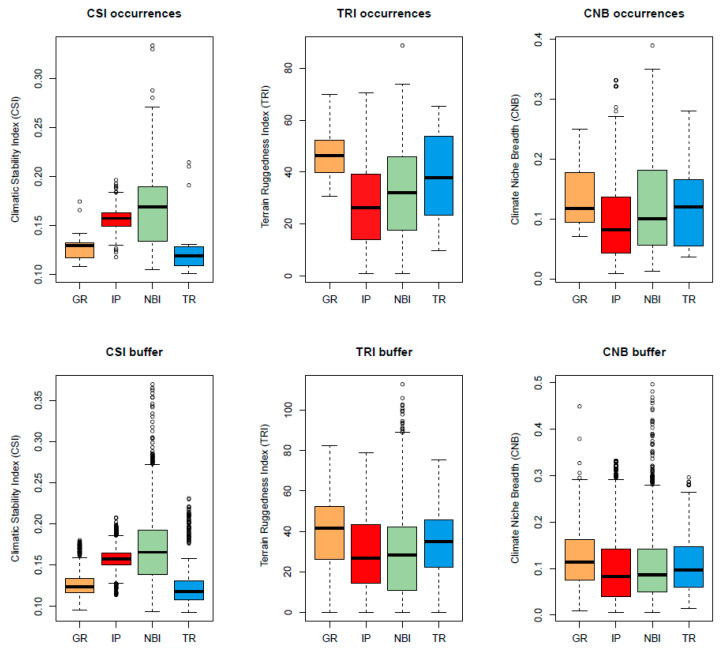
Values for the Climate Stability Index (CSI), the Terrain Ruggedness Index (TRI) and the Climate Niche Breadth (CNB) for each of the speciation centers, by (1) using only the species’ occurrence points and (2) using buffer areas of 10 km around each occurrence. GR, Greece; IP, Iberian Peninsula; NBI, Northern Balkans–Italy; TR, Turkey.

We checked the impact of climatic fluctuations and high-relief terrain with large environmental gradients as the second and third conditions of the MGH using the Climate Stability Index (CSI), the Terrain Ruggedness Index (TRI) and the Climate Niche Breadth (CNB). For both approaches (species occurrences and 10 km buffer areas), we found statistical differences for all comparisons regarding the Climatic Stability Index (CSI), with the only exception of Northern Balkans–Italy vs. Greece for the dataset of occurrences (Figure 4 and Supplementary Table S2). The largest values of CSI corresponded to Northern Balkans–Italy, followed by the Iberian Peninsula, Greece and Turkey. For the TRI, all comparisons were statistically significant for both occurrences and 10 km buffers; Greece was the most rugged area, followed by Turkey, Northern Balkans–Italy and the Iberian Peninsula (Figure 4 and Supplementary Table S2). Regarding the CNB, Greece showed the widest niche breadth, followed by Turkey, Northern Balkans–Italy and the Iberian Peninsula; however, while we detected statistical differences for all comparison pairs when taking 10 km buffers into account, for the occurrences, differences were only found when we compared the Iberian Peninsula vs. Northern Balkans–Italy and Greece (Figure 4 and Supplementary Table S2).

## 4. Discussion

### 4.1. Genetic Parameters and Speciation

The genetic structure resulting from our analyses reflects the presence of four geographic groups. However, there are some discordant populations in the Northern Balkans–Italy group, which show high levels of admixture with a predominance of the Iberian red genotype (Figure 2). The same Northern Balkans–Italy populations overlapped with some Iberian populations in the PCoA (Figure 3). The similarity of both genetic groups could be the result of hybridization of these populations with other species from subsect. *Centaurea*. In support of this hypothesis, we detected very high levels of admixture in the Northern Balkan–Italian populations [12], and hybridization was verified in the Iberian *C. costae* [13]. Genetic diversity is higher in the Iberian and Northern Balkans–Italy populations compared with the populations of the other speciation centers (Table 1). The high levels of genetic diversity in *Centaurea* could also be the result of hybridization followed by the repeated backcrossing of interspecific hybrids with one of the parents, as suggested by [48] for populations of *Centaurea podospermifolia* Loscos & J. Pardo. Certainly, increases in genetic diversity have frequently been reported in hybridizing populations in plants [49]. Both historical and recent interspecific gene flow in the Iberian Peninsula are extremely high, up to 10 times higher than in the other regions (Table 1), which support the extent of introgression within Iberian populations. High levels of gene flow are important to maintain high levels of genetic diversity within species, as alleles are less likely to be eliminated; due to high levels of gene flow, alleles tend to be shared in several populations, thus minimizing the effects of genetic drift. Only in the Iberian Peninsula, the historical levels of gene flow are theoretically capable of counteracting the effects of genetic drift (*Nm* > 1; [50]).

The Turkish and Greek populations, on the contrary, show the lowest levels of both historical and current gene flow, the lowest values of *H_e_* and the highest values of *F*_ST_, with the exception of Turkey for *F*_ST_, thus confirming that the species in these two speciation centers are strongly isolated (Table 1). According to the genetic data, we can conclude that speciation on the *Phalolepis* group follows two different models: allopatry by isolation in rugged mountains, exemplified by the cases of Greece and Turkey, and hybrid speciation as evidenced for the Iberian Peninsula. Speciation in the Northern Balkans–Italy region is closer to the Iberian model, even though gene flow is lower (Table 1).

### 4.2. Mountains and Speciation: Is the MGH Model Applicable to Our Case Study?

Although the elegant MGH model was formulated for explaining the factors accounting for the high biodiversity richness in the Tibeto-Himalayan region (THR), it was tested shortly after on 16 mountain systems around the world harboring important plant radiations [4]. With the help of general linear models (GLMs), the authors concluded that their model was supported, as the combination of three parameters was statistically significant for explaining plant species richness in mountain ranges: (1) net primary productivity (NPP), (2) elevation range, and (3) the change in the mean annual temperature (Δ*T*) between the LGM (Last Glacial Maximum, ca. 20,000 years BP) and today (the latter was taken as a rough proxy for climatic fluctuations for the whole Quaternary Period, i.e., the last 2.6 My). In addition, the authors also found that the range of elevation was positively correlated with a geodiversity index. However, Δ*T* showed a negative correlation with species richness; the authors [4] concluded that “mountain systems characterized by small elevation ranges and strong modifications of temperature […] appear to harbor less radiations”, and most radiations were identified in high-elevation mountains and weaker temperature oscillations. These patterns do not deny the second condition of the MGH hypothesis (the “species pump” effect associated with climatic instability) because, as noted by [4], most shifts in diversification rates of the radiations examined by these authors occurred much later than mountain-building processes. For the examples of the Tibetan–Hengduan Region and the Andes, this means that many radiations took place a few My after the orogenic movements of the Miocene, coinciding with the climatic changes of the Pliocene and Pleistocene Epochs. Alternatively, the negative correlation of species richness with Δ*T* could be due to the inability of this parameter (which measures the temperature differences between ca. 21,000 years ago and the present) to infer the temperature variations through the Pleistocene and Pliocene Epochs, when the vast majority of the 82 radiations examined by [4] took place; the cyclic temperature oscillations (Δ*T*) progressively increased from just about 1 °C in the early Pliocene to >6 °C in the late Pleistocene [51,52]. The recently developed CSI [22] uses 12 periods from the mid-Pliocene (3.3  My) to the present to estimate climate stability/instability. Thus, it could serve as a better option than Δ*T*_LGM–present_ for assessing the effect of climatic instability as the trigger of the species pump.

Our results for *Centaurea* in the Mediterranean mountains, which suggest a positive correlation between mountain ruggedness and speciation, fit well with the effects of Δ*T*_LGM–present_ observed by [4]. Although the CSI index used here combines six climatic variables instead of a single one, the regions with the maximum species diversity of subsect. *Phalolepis* (Turkey and Greece) have been the most climatically stable since 3.3 Mya, whereas the most climatically unstable regions (Iberian Peninsula, Italy–Northern Balkans) are the poorest in terms of species richness. The third condition of the MGH hypothesis (“high-relief terrain with environmental gradients”) is clearly met in the mountain systems of Turkey and Greece: these two regions showed the highest values for both TRI and CNB.

**Figure 5:**
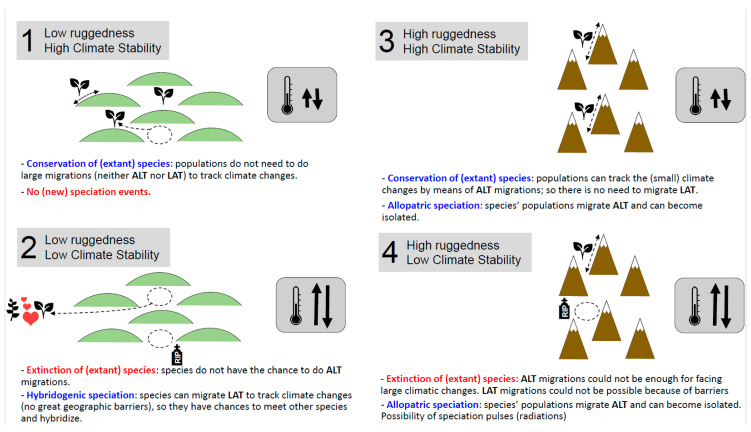
Possible scenarios resulting from the combination of various ruggedness and climatic stability conditions, and their consequences on the population dynamics and evolutionary patterns of plant species. Blue, net increase (or conservation) of species; red, net loss of species. ALT, altitudinally; LAT, latitudinally/longitudinally.

The topographical and climatic characteristics of the Iberian Peninsula and, at a smaller scale, Northern Balkans–Italy made them ideal for hybridogenic speciation; the combination of the strong climatic oscillations and the gentle topography forced and allowed, respectively, latitudinal shifts in species (Scenario 2 in Figure 5). Large migration movements caused species contact and favored hybridization; the special topography of the central part of the Iberian Peninsula would have enabled migrations of hundreds of km, which would have ensured the integrity of, e.g., *Centaurea alba* [13]. The very low number of species of subsect. *Phalolepis* in this region is not unexpected and is likely due to the limited speciation processes via hybridization coupled with putative extinction processes; some species, particularly narrow endemics, would have not had the chances for altitudinal migrations in the low-elevation Meseta (Figure 5).

Greece and Turkey, in contrast, became relatively important speciation centers of subsect. *Phalolepis*, with events of allopatric speciation shown by the genetic markers, likely the consequence of a very rugged terrain providing geographical barriers. The speciation of *Centaurea* does not constitute a radiation in any way because the nuclei of maximum diversity in Anatolia and Greece comprise no more than 30 species, the mountains have only moderate altitudes of generally 2000–3000 m, and the climate was probably not unstable enough. Nevertheless, within the context of the Mediterranean Basin, both the Greek and Turkish mountain ranges may superficially resemble the THR and the Andes [4]. These two very high cordilleras are examples of “mountain systems with the largest elevational ranges and stronger overlap between today’s and LGM temperature profiles and are also those where most radiations were identified” [4]. Rugged mountains with large altitudinal gradients allow species to survive climate changes by moving up and down; if the climate oscillations are not large, the required altitudinal shifts are not so extensive, which would help populations to keep populations large enough to avoid local extinction (Scenario 3 in Figure 5). If high ecological gradients are added to the equation (herein measured though the CNB), then plant species could easily find favorable pockets for survival (e.g., “microrefugia” sensu [53]) while stimulating ecological speciation [54,55]. Allopatric and ecological speciation are not mutually exclusive; thus, they can contribute to the patterns of high species richness in topographically and ecologically complex mountains.

The patterns of *Centaurea* subsect. *Phalolepis* in the Turkish and Greek mountain ranges greatly resemble those of Scenario 3 in Figure 5: the dual role of mountains as species cradles due to allopatric speciation, and as species museums due to the occurrence of (micro)refugia. This feature of many mountains has undoubtedly attracted much interest on a conservation basis [56,57,58], as their efficient preservation would allow for the protection of those ecological and evolutionary processes that create and maintain biodiversity due to the inherent topographic and environmental heterogeneity.

### 4.3. Climate Niche Breadth (CNB): A New Index for Representing Climate Niche Breadth at the Global Scale

Here, we have implemented a new parameter that has allowed us to test environmental gradients against the rate of allopatric speciation in *Centaurea* subsect. *Phalolepis*. Although the CNB only includes climatic aspects, it has the paramount advantage that data are available for the whole Earth (the bioclimatic variables of WorldClim); therefore, users could select a desired subset of the climatic variables (or all 19 combined ones) and create their “own” CNB for a given region (or the whole planet). Through Figshare (https://doi.org/10.6084/m9.figshare.20863528), researchers can access two sets of data records: (1) SD-based maps of individual bioclimatic variables, which contain a set of raster layers (19, one for each variable); (2) a SD-based map for the 19 bioclimatic variables combined; and (3) the scripts that allow them to combine the raster layers according to the user’s preferences to generate a customized CNB.

**Figure 6:**
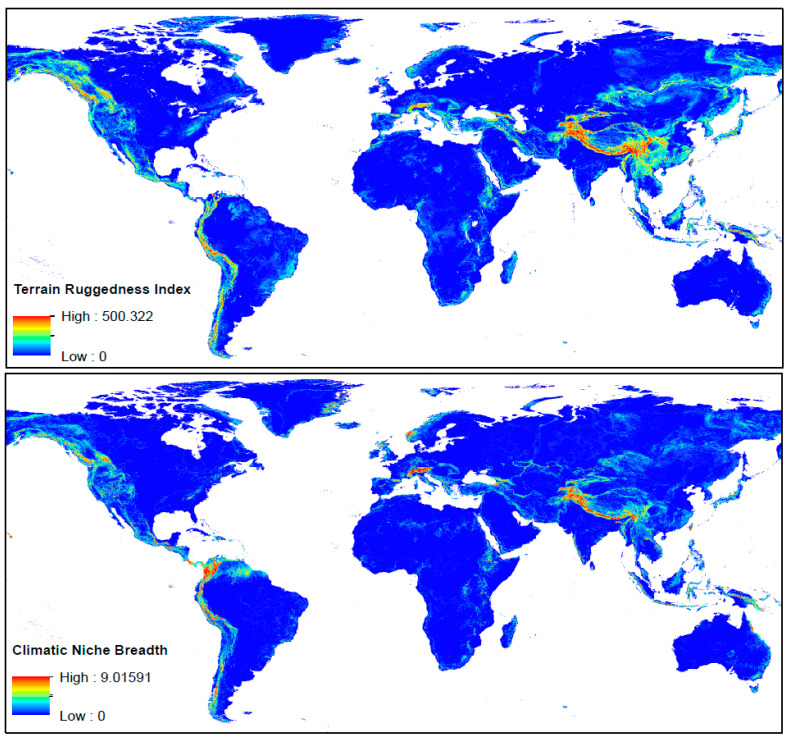
Maps of the Terrain Ruggedness Index (TRI) and the Climate Niche Breadth (CNB) values at a 2.5 arc-min grid resolution for the whole planet. Colors range from blue for low values to red for high values; the color gradient shows equal interval breaks.

As expected, there is a large positive correlation between TRI and CNB: *r* = 0.796 for our study area. By definition, rugged terrains provide a wide array of climatic conditions; thus, it is not rare that rugged mountains have traditionally been linked to glacial refugia, as species were able to find local microclimates and thus persisted [59,60]. Despite this relationship between TRI and CNB, the *r* values are well below 1, which indicates that the ruggedness alone cannot fully explain the breadth of climatic niches. This correlation is reduced on a global level (*r* = 0.634); if we compare the global maps of TRI and CNB (Figure 6), there are some important differences. For instance, the Colombian Andes, which is one the regions of the world with the highest levels of CNB, has only moderate values for TRI, and the same is applicable to the Alps. Conversely, some areas with high/moderate values of TRI show moderate/low values for CNB, particularly in central, east (including the Russian Far East) and southeast Asia (Figure 6). The differences can be very high at a more local scale; examples include some parts of northern Queensland (Australia) and Pantepui in the Guiana Region, with the values of CNB much higher than those of TRI (Figure 6). In the case of the Pantepui region, this discrepancy is easy to explain: the topography is relatively simple, and the landscape is characterized by the tabular mountains or “tepuis”, which are typically 2000–2600 m high, emerging from the lowland savannas, which are generally 100–400 m high [61], but these large vertical gradients offer a wide array of microclimates.

## 5. Concluding Remarks

Our analyses of *Phalolepis* highlighted the crucial role of the mountains in both the genesis and the preservation of species diversity. Ruggedness emerged as the dominant factor, but all the conditions indicated by [3] for the Mountain–Geobiodiveristy Hypothesis, namely the presence of lowland, montane and alpine zones; climatic oscillations that enable mountains to act as species pumps; and high-relief terrain with environmental gradients, must be present for true radiation. In the Iberian Peninsula, with the lowest ruggedness and the presence of a high plateau connecting mountain ranges, the diversity was very low and speciation was hybridogenic. In the other Mediterranean mountains, high ruggedness favored allopatric speciation; however, climatic stability was high, which would have impeded true radiation and, at the same time, reinforced their role as refugia during periods of glaciation. This pattern of species evolution stresses the need for special protection of the montane ranges in the Mediterranean Region.

## Figures and Tables

**Table 1:** Results of the analyses of Terrain Ruggedness Index (TRI), Climate Stability Index (CSI) and Climate Niche Breadth; and genetic parameters and gene flow of the studied species.

Regions	Species n°	TRI **	CSI **	CNB **	Genetic Diversity (*H_e_*)	Genetic Differentiation (*F_ST_*)	Past Gene Flow (*Nm*)	Recent Gene Flow (*m*)
**Turkey**	9(7) *	38.53/33.71	0.128/0.129	0.124/0.108	0.580	0.198	0.466	0.0044
**Greece**	22(7) *	47.89/38.83	0.129/0.130	0.136/0.123	0.587	0.243	0.534	0.0024
**Italian Peninsula/Northern Balkans**	10(6) *	32.45/28.69	0.172/0.171	0.125/0.103	0.662	0.232	0.645	0.0084
**Iberian Peninsula**	2(2) *	27.86/29.41	0.157/0.157	0.096/0.099	0.645	0.176	7.475	0.0526

(*) In parenthesis, species included in this study. (**) The first value was calculated by each occurrence of the studied species, and the second value was calculated by a buffer area of 10 km.

## Data Availability

All the data used in this paper are already available; please refer to [10,11,12,13].

## Supplementary Materials

The following supporting information can be downloaded at: https://www.mdpi.com/article/10.3390/plants12010011/s1, Supplementary Table S1: Voucher information. Supplementary Figure S1: Results of the Evanno test [28] for the best *K*. Supplementary Table S2: *p* values for the comparisons among the four speciation centers regarding the Climate Stability Index (CSI), the Terrain Ruggedness Index (TRI) and the Climate Niche Breadth (CNB).
