# Supplementary material for: Centaurea Subsect. Phalolepis (Compositae, Cardueae): A Case Study of Mountain-Driven Allopatric Speciation in the Mediterranean Peninsulas
Source: Plants (Basel). 2022 Dec 20;12(1):11. doi: 10.3390/plants12010011 (PMC9823864; doi:10.3390/plants12010011)
Supplement: Supplementary file 1 [file plants-12-00011-s001.zip › Suppl. Table S1.pdf]

Species	Plant Locality	Coordinates
Centaurea alba L. subsp. *alba* var. *alba*	Spain, Segovia: Puerto de Guadarrama, on southern side, 10.07.2009, *Hilpold 20093100 & Vilatersana* (BC) **[ALB1a]**<br>Spain, Soria: Hoz de arriba, peña del Santo, 25.06.2010, *Garcia-Jacas & Romaschenko 641* **[ALB2a]**<br>Spain, Ávila: Sierra de Gredos, La Adrada, 2011, *Hilpold 20113194* **[ALB3a]**	40°42'37"N, 4°8'23"W<br>41°23'56.4", 3°7'48"W<br>40°20'2.4", 4°38'6"W
Centaurea alba L. subsp. *alba* var. *latronum* (Pau) E. López & Devesa	Spain, Ávila: El Barco de Ávila, along the road to Los Llanos de Tormes, about 2.5 km after El Barco, 09.07.2009, *Hilpold 20093089 & Vilatersana* (BC) **[ALBl]**	40°20'2,5"N, 5°30'53.3"W
Centaurea alba L. subsp. *alba* var. *macrocephala* Pau	Spain, Cádiz: between Arcos and Bornos, 21.07.2008, *Garcia-Jacas & Susanna 2689* (BC) **[ALBm]**	36°48'54N", 5°48'47"W
Centaurea alba L. subsp. *aristifera* (Pau) E. López & Devesa	Spain, Zaragoza: Embid de la Ribera going to Bilbilis, 07.07.2012, *Garcia-Jacas & Susanna 2783* (BC) **[ARI]**	41°24'46.8"N, 1º3 6'25.2"W
Centaurea alba L. subsp. *strepens* (Hoffmanns. & Link) Rocha Afonso	Portugal: by coast of Tejo, N of Tramagal and W Abrantes, 06.07.2009, *Hilpold 20093068, Garcia-Jacas & Vilatersana* (BC) **[STR]**	39°28'16"N, 8°15'7"W
Centaurea alba L. subsp. *tartesiana* Talavera	Spain, Huelva: Sierra de Aracena, between Los Marines and Fuenteheridos, 04.05.2008, *Barres 74 & Molero* (BC) **[TAR1]**<br>Spain, Badajoz: Calera de León, Sierra de Tentudia, on road BA-109, km 12.5, 03.09.2016, *F. Márquez s. n.* **[TAR2]**	37°54'4.7"N, 6°39'16.2"W<br>38º3'27"N, W6º20'4.88"W
Centaurea amaena Boiss. & Balansa	Turkey, Kayseri: Yılan Dağı, Kayalık slopes, 1141 m, 23.06.2009, *Ertuğrul, Tugay*, *Uysal* & *Susanna 2721* (BC) **[AMA]**	38°42.874'N, 35°25.064'E
Centaurea antalyensis H.Duman & A.Duran	Turkey, Antalya: Akseki, between Murtiçi and Güzelsu, 1036 m, 25.06.2009, *Ertuğrul, Tugay*, *Uysal* & *Susanna 2729* (BC) **[AN]**	36°54.493'N, 31°48.939'E
Centaurea aspromontana Brullo, Scelsi & Spamp	Italy, Calabria: road from S. Cristina Aspromonte to Oppidum Mamertina, 26.05.2008, *Hilpold, Garcia-Jacas & Vilatersana 1137* (BC) **[ASP]**	38°15'2"N, 15°58'59"E

C. brunnea (Halácsy) Halácsy	Greece, Nomos Prevezis, Eparchia Nikopoleos-Pargas: c. 2.7 km N from the village of Kamarina along road to Kriopigi, 679 m, 02.08.2009, *Garcia-Jacas, Karamplianis & Susanna 2750* (BC) **[BRU1]**<br>Greece, Nomos Prevezis, Eparchia Nikopoleos-Pargas: c. 0.5 km S of the village of Kriopigi, 577 m, 03.08.2009, *Garcia-Jacas, Karamplianis & Susanna 2752* (BC) **[BRU2]**	39°08.955'N, 20°40.858'E<br>39°08.862'N, 20°39.822'E
Centaurea cadmea Boiss.	Turkey, Denizli: Honaz, on the castle, 689 m, 20.06.2009, *Ertuğrul, Tugay, Uysal & Susanna 2708* (BC) **[CA1]**<br>Turkey, Zonguldak: Devrek, Eğerci, Taşlık village, 200 m, 03.06.2009, *Tugay 5761* (KNYA) **[CA2]**<br>Turkey, Bartın: Ulus-Ulukaya Şelaesi, Kaya road, 241 m, 22.06.2009, *Ertuğrul, Tugay, Uysal & Susanna 2715* (BC) **[CA3]**	37°45.052'N, 29°15.995'E'<br>41°5.752'N, 31°48.982'E<br>41°35.356'N, 32°41.110'E
C. chrysocephala Phitos & T. Georgiadis	Greece, Nomos Trikalon, Eparchia Kalampakas: W-facing vertical rocks at the entrance to the monastery of Varlaam (Meteora), 532 m, 01.08.2009, *Garcia-Jacas, Karamplianis & Susanna 2736* (BC) **[CHR3]**<br>Greece, Nomos Trikalon, Eparchia Kalampakas: W-facing slopes near the entrance of the monastery of Holy Trinity (Meteora), 494 m, 01.08.2009, *Garcia-Jacas, Karamplianis & Susanna 2737* (BC) **[CHR4]**<br>Greece, Nomos Trikalon, Eparchia Kalampakas: Mt. Trigia, c. 1 km E of the village of Klinovo, 816 m, 02.08.2009, *Garcia-Jacas, Karamplianis & Susanna 2742* (BC) **[CHR1]**<br>Greece, Nomos Trikalon, Eparchia Kalampakas: Mt. Neraida, c. 2 km SW from Pertouli Ski Center to Neraidochori, 1153 m, 02.08.2009, *Garcia-Jacas, Karamplianis & Susanna 2747* (BC) **[CHR5]**<br>Greece, Nomos Trikalon, Eparchia Kalampakas: c. 1 km N main road Kastania-Kalampaka, 721 m, 02.08.2009, *Garcia-Jacas, Karamplianis & Susanna 2749* (BC) **[CHR2]**	39°43.574'N, 21°37.871'E<br>39°42.790'N, 21°38.228'E<br>39°40.037'N, 21°27.634'E<br>39°32.165'N, 21°27.321'E<br>39°43.050'N, 21°22.980'E
Centaurea costae Willk. var. *costae*	Spain, Lleida: Montsec, on northern side, 1 km ESE Cellers, river bed Barcedana, 01.07.2010, *Hilpold 20104057 & López-Alvarado* (BC) **[COSc]**	42°3'36"N, 0°54'4"E

Centaurea costae Willk. var. *maluqueri* Font Quer	Spain, Huesca: Montsec, between castle Viacamp and road Lleida-Vielha (N230), 0,2 - 0,5 km NW castle, 23.05.2008, *Hilpold s.n.* (BC) **[COS1ma]**<br>Spain, Lleida: Pallars Sobirà, allong road from Sellui to Ancs, 1.4 km NNW Sellui, 14.07.2010, *Hilpold 20104074 & Vogel* (BC) **[COS2ma]**	42°7'52"N, 0°36'43"E<br>42°41'47"N, 1°0'48"E
Centaure costae Willk. var. *montsicciana* Pau & Font Quer	Spain, Lleida: Montsec, on northern side, 1 km ESE Hostal Roig, 1 km NW Pas Nou, 01.07.2010, *Hilpold 20104063 & López-Alvarado* (BC) **[COSmo]**	42°2'3"N, 1°1'17"E
Centaurea deusta Ten.	Greece, Nomos Fthiotidos, Eparchia Fthiotidos: Mt. Iti near Kastania, 850 m, 07.08.2012, *Constantinidis*, *Garcia-Jacas & Susanna 2788* (BC) **[DEU1]**<br>Greece, Nomos Magnisias, Eparchia Volou: Mt. Pilio between Volos and Drakia, 911 m, 08.08.2012, *Constantinidis, Garcia-Jacas & Susanna 2790* (BC) **[DEU2]**<br>Bulgaria, Stara Zagora: Tazha village, 650 m, 6.08.2012, *Bancheva*, *Garcia-Jacas & Susanna 2784* (Susanna pers. herb.) **[DEU3]**<br>North Macedonia, Mavrovo and Rostuša: between Galičnik and Mavrovo, 7.07.2012, *Janaćković* (Susanna pers. herb.) **[DEU4]**<br>North Macedonia, Ohrid: Galičica, 5.07.2012, *Janaćković* (Susanna pers. herb.) **[DEU5]**<br>Serbia, Zlatibor: road from Zlatibor to Semegnjevu, , 21.06.2012, *Tomović* (Susanna pers. herb.) **[DEU6]**<br>Montenegro, Budva: above Budva around the tunnel, 7.06.2012, *Novaković* (Susanna pers. herb.) **[DEU7]**<br>Croacia, road from Omiš to Vukosavić, along the Cetina river, 24 m, 11.07.2011, *López-Pujol & Massó 6* **[DEU8]**<br>Italy, Umbria: hill to 4 km SE Norcia, along road to Marche, 820 m, 24.8.2011, *Hilpold 20115006 & Sánchez-Meseguer* (Susanna pers. herb.) **[DEU9]**<br>Italy, Campania: N of San Gregorio Matese, road to Lago Matese, 880 m, 28.7.2009, *Hilpold 20094008 & Granitto* (Susanna pers. herb.) **[DEU10]**<br>Italy, Basilicata: Potenza, Pollino, National Park of Pollino, 25.5.2008, *Hilpold, Garcia-Jacas & Vilatersana 1131* (BC) **[DEU11]**<br>Italy, Toscana: Grosseto, Mt. Amiata, about 2 km E of Castel del Piano, road to Amiata, 850 m, 25.8.2011, *Hilpold 20115017 & Sánchez-Meseguer* (Susanna pers. herb.) **[DEU12]**	38°51'15"N, 22°12'3"E<br>39°23'4"N, 23°3'26"E<br>42°32'39.47"N, 25°4'49"E<br>41°37'17.8"N, 20°42'27"E<br>40°57'15.7"N, 20°48'59.8"E<br>43°43'58.66"N, 9°40'18.80"E<br>42°16'47.04"N, 18°49'20.40"E<br>43°26'57.58"N, 16°42'25.48"E<br>42°46'3"N, E13°8'5"E<br>41°23'15"N, E14°23'11"E<br>39°56'15"N, E16°8'13"E<br>42°53'25"N, E11°34'7"E

C. heldreichii Halácsy	Greece, Nomos Etolias-Akarnanias, Eparchia Mesolongiou: E of Krioneri settlement, on the rocky slopes and cliffs of Varasova mountain and also on the lower parts of the mountain with conglomerate, 2-10 m, 09.05.2004, *Constantinidis 10993* (ATHU) **[HEL]**	8°20.618'N, 21°35.944'E
Centaurea ionica Brullo	Italy, Calabria: Gerace, santuary below the village, 25.05.2012. *Garcia-Jacas & Susanna 2769* (Susanna pers. herb.) **[ION1]**<br>Italy, Calabria: road from Pazzano to Monte Stella (SS110) about 1 km WSW Pazzano, 31.05.2008, *Hilpold*, *Garcia-Jacas & Vilatersana 1191* (BC) **[ION2]**	38°16'27''N, 16°13'12''E<br>38°28'20''N, 16°26'35''E
C. litochorea T. Georgiadis & Phitos	Greece, Nomos Larissis, Eparchia Tirnavou: Mt Kato Olimbos, at the summit area of Analipsi, below the telecommunication antennas, NW of the village of Kallipefki, 1359 m, 30.07.2009, *Garcia-Jacas, Karamplianis & Susanna 2731* (BC) **[LIT1]**<br>Greece, Nomos Larissis, Eparchia Tirnavou: Mt Kato Olimbos, near the summit area E of the village of Kallipefki, along the main path from the chapel of Metamorphosis to the village of Kallipefki, 1441 m, 31.07.2009, *Garcia-Jacas, Karamplianis & Susanna 2732* (BC) **[LIT2]**	39°57.943'N, 22°26.763'E<br>39°57.477'N, 22°29.487E'
Centaurea luschaniana Heimerl ex Stapf	Turkey, Antalya: road Korkuteli-Elmalı, Karamanbeli, 1337 m, 20.06.2009, *Ertuğrul, Tugay*, *Uysal* & *Susanna 2705* (BC) **[LU]**	36°56.681'N, 30°09.617'E
Centaurea lycaonica Boiss. & Heldr.	Turkey, Konya: Seydiehir road, 1595 m, 25.06.2009, *Ertuğrul, Tugay*, *Uysal & Susanna 2728* (BC) **[LY]**	37°45.054'N, 32°04.529'E
Centaurea lycia Boiss.	Turkey, Antalya: Korkuteli road, 20 km, 768 m, 20.06.2009, *Ertuğrul*, *Tugay*, *Uysal & Susanna 2702* (BC) **[LYC1]**<br>Turkey, Antalya: Saklıkent road, Koz Dağı, Taşlı slopes, 1198 m, 19.06.2009, *Ertuğrul, Tugay*, *Uysal* & *Susanna 2700* (BC) **[LYC2]**	37°00.045'N, 30°29.409'E<br>36°53.511'N, 30°22.153'E
C. messenicolasiana T. Georgiadis, Dimitrellos & Routsi	Greece, Nomos Karditsis, Eparchia Karditsis: c. 2 km S and E of Messenikolas village, along the road to Karditsa, 597 m, 01.08.2009, *Garcia-Jacas, Karamplianis & Susanna 2739* (BC) **[MES]**	39°20.301'N, 21°45.813'E
Centaurea nobilis (Groves) Brullo	Italy, Puglia: 4 km SSE Otranto, Punta Palascia, about 0.1 km NNW of Faro, 01.6.2008, *Hilpold, Garcia-Jacas & Vilatersana 1197* (BC) **[NOB]**	40°6'30''N, 18°31'8''E

Centaurea pentadactyli Brullo, Scelsi & Spamp.	Italy, Calabria: Pentedattilo village, on the eastern side of the rocks, 31.05.2008, *Hilpold, Garcia-Jacas & Vilatersana 1183* (BC) **[PEN1]**<br>Italy, Calabria: Bova, road to Bova Marina, 5 km from the junction of road 106, 25.05.2012, *Garcia-Jacas & Susanna 2768* (Susanna pers. herb.) **[PEN2]**	37°57'16"N, 15°45'48"E<br><br>37°57'28"N, E15°56'54"
C. princeps Boiss. & Heldr.	Greece, Nomos Evritanias, Eparchia Evritanias: Mt. Timfristos, at the NW area of the main summit of Koumbi, 1468 m, 04.08.2009, *Garcia-Jacas, Karamplianis & Susanna 2756* (BC) **[PRI1]**<br>Greece, Nomos Evritanias, Eparchia Evritanias: Mt. Timfristos, SE parts of the mountain, c. 8.0-8.5 km E of Karpenisi, c. 1200-1240 m, 26.07.2008, *Constantinidis s.n. & Karamplianis* (ATHU) **[PRI2]**	38°56.240'N, E21°51.732'E<br><br>38°55.044'N, E21°51.245'E
Centaurea scillae Brullo	Italy, Calabria: ca.1.5 km SW of Bagnara on the road to Favazzina, km 505, 26.05.2008, *Hilpold, Garcia-Jacas & Vilatersana 1140* (BC)	38°16'28" N, 15°47'7"E
Centaurea wagenitzii Hub.-Mor.	Turkey, Antalya: Adrasan bay, south end, 14 m, 19.06.2009, *Ertuğrul, Tugay*, *Uysal & Susanna 2697* (BC) **[WA1]**<br>Turkey, Antalya: Adrasan, north end, 20 m, 19.06.2009. *Ertuğrul, Tugay*, *Uysal* & *Susanna 2698* (BC) **[WA2]**	36°17.817' N, 30°28.455' E<br><br>36°18.770'N, 30°27.812'E
