# Supplementary material for: Centaurea Subsect. Phalolepis (Compositae, Cardueae): A Case Study of Mountain-Driven Allopatric Speciation in the Mediterranean Peninsulas
Source: Plants (Basel). 2022 Dec 20;12(1):11. doi: 10.3390/plants12010011 (PMC9823864; doi:10.3390/plants12010011)
Supplement: Supplementary file 1 [file plants-12-00011-s001.zip › Suppl. Table S2.pdf]

Supplementary Table S2. *p*-values for the comparisons between the four speciation centers regarding Climate Stability Index (CSI), Terrain Ruggedness Index (TRI) and Climate Niche Breadth (CNB).

Species Occurences

CSI			
Wilcoxon–Mann–Whitney test	Iberian Peninsula	N Balkans-Italy	Turkey
Greece	8,07E-09	0,0001394	7,73E-02
Iberian Peninsula		0,01484	3,74E-10
N Balkans-Italy			5,29E-06

TRI			
Wilcoxon–Mann–Whitney test	Iberian Peninsula	N Balkans-Italy	Turkey
Greece	9,56E-07	0,0006334	8,16E-02
Iberian Peninsula		0,02942	2,52E-03
N Balkans-Italy			1,20E-01

CNB			
Wilcoxon–Mann–Whitney test	Iberian Peninsula	N Balkans-Italy	Turkey
Greece	0,001651	0,1914	0,3668
Iberian Peninsula		0,003664	0,7936
N Balkans-Italy			0,9657

10-km buffer areas

CSI			
Wilcoxon–Mann–Whitney test	Iberian Peninsula	N Balkans-Italy	Turkey
Greece	2,20E-16	2,20E-16	5,43E-06
Iberian Peninsula		2,20E-16	2,20E-16
N Balkans-Italy			2,20E-16

TRI mean			
Wilcoxon–Mann–Whitney test	Iberian Peninsula	N Balkans-Italy	Turkey
Greece	2,20E-16	2,20E-16	3,22E-05
Iberian Peninsula		0,003382	9,04E-08
N Balkans-Italy			2,79E-08

CNB			
Wilcoxon–Mann–Whitney test	Iberian Peninsula	N Balkans-Italy	Turkey
Greece	4,21E-14	4,98E-11	0,0004549

Iberian Peninsula	1,49E-01	7,19E-06
N Balkans-Italy		0,001482
