# Supplementary material for: Centaurea Subsect. Phalolepis (Compositae, Cardueae): A Case Study of Mountain-Driven Allopatric Speciation in the Mediterranean Peninsulas
Source: Plants (Basel). 2022 Dec 20;12(1):11. doi: 10.3390/plants12010011 (PMC9823864; doi:10.3390/plants12010011)

Fig. S1 Screening for the most likely value of *K*. Number of groups (*K*) with non-hierarchical *K*-means clustering (Evanno et al., 2005), and the smallest *K* after the log probability of data [ln Pr(*X*|*K*)] values reached a plateau (Pritchard et al., 2000).

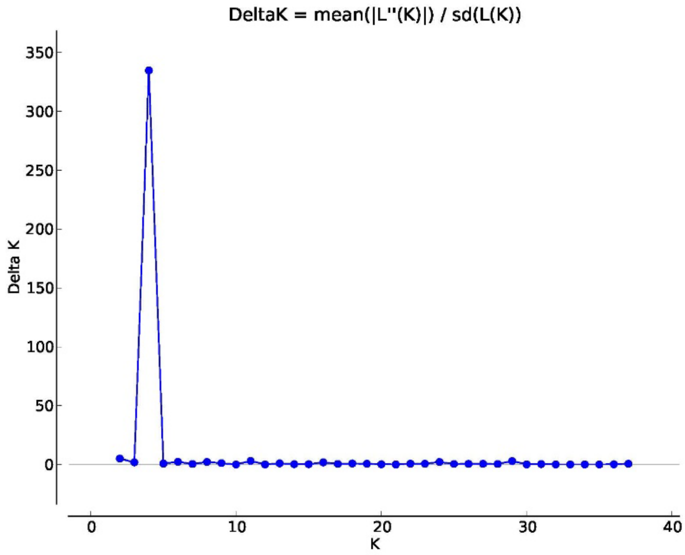

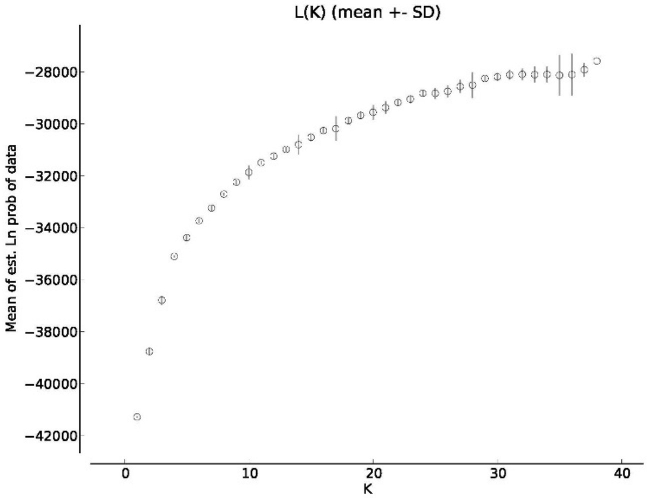

Supplement: Supplementary file 1 [file plants-12-00011-s001.zip › Supplementary Figure S1.pdf]
